# Is there a diurnal variation of COVID-19 patients warranting presentation to the health centre? A chronobiological observational cross-sectional study

**DOI:** 10.1080/07853890.2022.2136399

**Published:** 2022-10-29

**Authors:** Mohamed Romdhani, Sakthikumar Vedasalam, Amine Souissi, Mohamed Saifeddin Fessi, Amit Varma, Morteza Taheri, Amine Ghram, Abdulla Al Naama, Bessem Mkaouer, Helmi Ben Saad, Ismail Dergaa

**Affiliations:** a Research Unit: Physical Activity, Sport, and Health, UR18JS01, National Observatory of Sport, Tunis, Tunisia; b Motricité-Interactions-Performance, MIP, UR4334, Le Mans Université, Le Mans, France; c Primary Health Care Corporation, Doha, Qatar; d Sports Medicine, Universite de Sousse, Faculte de Medecine de Sousse, Sousse, Tunisia; e Department of Sport Sciences, Imam Khomeini International University, Qazvi, Iran; f Healthy Living for Pandemic Event Protection (HL – PIVOT) Network, Chicago, IL, US; g Laboratoire de Recherche (LR12SP09) “Insuffisance cardiaque” Sousse, Faculté de Médecine de Sousse, Hôpital Farhat HACHED, Université de Sousse, Sousse, Tunisie

**Keywords:** Chronobiology, immunity, pandemic, RT-PCR, SARS-CoV-2, time of day, viral infection

## Abstract

**Background:**

The circadian clock regulates the function of the immune system, the replication of viruses, and the magnitude of infections. The aim of this study was to analyse whether hospital attendance in Coronavirus disease 2019 (COVID-19) patients presents a diurnal variation.

**Methods:**

Data from the electronic medical records of 1094 COVID-19 patients who presented to a Health Centre in Qatar during the month of July 2020 was retrospectively analysed. The following demographic (i.e. time of day (TOD), sex, age), clinical (i.e. cycle threshold (CT), temperature, oxy-haemoglobin saturation and resting heart-rate), biochemical (i.e. uraemia, glycaemia and albuminia) and haematological (i.e. leukocytes, erythrocytes ad platelets) parameters were collected.

**Results:**

Univariate analysis showed a significant effect of TOD on hospital admission (*p* < 0.001), with patients attending the health care centre more during the active behavioural phase (08h00-00h00) compared to the resting phase (00h00-08h00). COVID-19 infection blunted the circadian rhythms of core body temperature, neutrophils and leukocytes family and shifted the circadian rhythms of resting heart-rate and uraemia. Correlation analysis showed a near perfect negative correlation between the age of patients and the TOD (*r*=–0.97), with older patients attending the care centre earlier during the day.

**Conclusion:**

COVID-19 infection affected the circadian rhythms of the host through disrupting the circadian rhythms of core temperature and innate immunity mediators. Old patients attend the health care centre earlier compared to younger ones. However, CT during polymerase chain reaction-test was unaffected by the TOD, which limits the conclusion that COVID-19 viral infection exhibits diurnal variation.

## Introduction

The Coronavirus disease 2019 (COVID-19), which has been declared a public health epidemic of global outrage by the World Health Organisation (WHO), is among the most shocking diseases in recent years [1]. There were nearly 620 M laboratory reported cases and over 6.5 M deaths worldwide as of 26 September 2022 (https://www.worldometers.info/coronavirus/). Even though COVID-19 can affect people of all ages, older people are at a higher risk of deleterious consequences, as well as a greater fatality rate [2,3]. For instance, in a cohort of 8516 American veterans, hospital admission during the high load period of intensive care units was associated with two-fold mortality compared to patients treated during the low load period [4]. Understanding the origins of this temporal distribution will increase the efficiency of health care intervention [1]. However, little is known about the circadian distribution of COVID-19 patient admissions.

Almost all physiological and behavioural functions fluctuate over the 24-h cycle. This cycle, namely the circadian rhythm, is generated by the suprachiasmatic nucleus in the hypothalamus and coordinated by the peripheral circadian clock located in distal tissue by way of hormones, clock genes and body temperature [5,6]. Circadian rhythmicity is an evolutionarily conserved pathway that serves to adapt the organism to the 24-h environmental day [7,8]. Because the exposition of the host to pathogens varies across the 24-h cycle, the circadian clock anticipates environmental daily changes in order to optimize the immune response [8–10]. Several types of illness exhibit diurnal variation [11,12]. For instance, respiratory diseases (viral and allergic rhinorrhoea, nocturnal asthma, and chronic pulmonary obstructive disease) display circadian variation with symptoms and/or gravity requiring hospital attendance occurring during the night [11]. Consequently, mediators of the immune system have been shown to exhibit diurnal variations in the blood and tissues, including immune cells, antibodies, cytokines, chemokines and hormones [10]. During the active phase, immune cells migrate to the peripheral tissues in a major anti-microbial response, and the circulating number of immune cells increases during the night [13].

Clinical manifestations of COVID-19 seem to be comparable, with some differences, to influenza [14]. As previous research confirmed that influenza infections and the severity of symptoms are affected by the time of the day (TOD) [11], it seems prudent to study the effect of TOD on COVID-19 patient hospital attendance. Importantly, a recent study reported a diurnal variation in the positive severe acute respiratory syndrome coronavirus 2 (SARS-CoV-2) test, with a peak around 14h00 [15]. It has been consistently reported that circadian rhythm regulation differs between males and females [16], with females displaying smaller diurnal amplitude of temperature [17] and higher diurnal amplitude of melatonin [7] compared to males. Further, males and females sleep differently [18] and respond differently to sleep deprivation [17–19]. Indeed, the COVID-19 and its associated lockdown affected the sleep-wake cycle [20–22], with female being more disrupted than males [23–25]. However, little is known concerning the existence of a sex-based time course of COVID-19 patients’ hospital attendance. Both physiological sleep and endogenous melatonin secretion, which present sex-based differences, play an important role in innate immunity regulation [26,27]. The observation that COVID-19 patients reported highly disturbed sleep-wake cycles [24] may be indicative of an infection-based circadian disruption. In addition, older people show reduced circadian flexibility and could be more affected when facing a circadian disrupter [16].

Therefore, the current study aimed to investigate the diurnal distribution of hospital attendance in COVID-19 positive patients. The second aim was to study the sex and age differences in COVID-19 positive patients attending hospital. Based on the existing literature, it was hypothesized that the COVID-19 could also exhibit diurnal variation in hospital attendance, which may be affected by sex and age.

## Patients and methods

### Study design

In this study, we retrospectively analysed data from the electronic medical records of 1049 COVID-19 positive patients who had presented to Rawdat Al Khail Health Centre, Doha, Qatar (RAK-HC) during the period between 1 July and 31 July 2020. This study was approved by the institutional review board at Primary Health Care Corporation (PHCC-Ref No. PHCC/DCR/2020/08/091) in the spirit of the Helsinki Declaration (64th World Medical Association General Assembly, Fortaleza, Brazil, October 2013). All respondents provided written consent for anonymous data use for research purposes and publications. The data was handled in an anonymous way and in line with the ‘General Data Protection Regulation’ (gdpr-info.eu).

### Patient population and data collection

This retrospective observational study included all COVID-19 positive patients who presented to RAK-HC during the month of July 2020. The parameters looked at were time of registration of the patients, when they presented to RAK-HC for COVID-19 testing, and demographic data (i.e. age and sex) of patients. Patients presenting to RAK-HC with symptoms of probable COVID-19 underwent oropharyngeal and nasopharyngeal swabs by trained healthcare professionals to test for SARS-Cov-2 by reverse transcription polymerase chain reaction (RT-PCR) testing at the laboratory in Hamad Hospital, which is the government designated testing laboratory in Qatar. Only COVID-19 positive patients were included in this study.

Demographic (i.e. TOD, age and sex), clinical [i.e. cycle threshold (CT), core body temperature, resting heart-rate and oxy-haemoglobin saturation (SpO_2_)], biochemical (i.e. albumin, plasma glucose and urea) and haematological (i.e. erythrocytes, leukocytes, neutrophils and platelets) data were collected from the electronic medical records after all patient identifiable data being removed as per the research study requirements.

### Statistical analysis

The statistical tests were processed in GraphPad Prism 6 (GraphPad Software, San Diego, CA). The quantitative data was expressed as mean ± standard deviation (SD). Comparison between different TOD [six time frames (00h00-04h00; 04h00-08h00; 08h00-12h00; 12h00-16h00; 16h00-20h00 and 20h00-00h00)] was assessed by a One way analysis of variance (ANOVA). Sex-based comparison at different TOD was assessed by a Two way ANOVA [six time frames (00h00-04h00; 04h00-08h00; 08h00-12h00; 12h00-16h00; 16h00-20h00 and 20h00-00h00) * sex (male/female)]. Once the ANOVA indicates a significant effect or interaction, a Bonferroni post-hoc test was performed to compare data by pair. Pearson product-moment correlation ‘Pearson *r*’ was performed between the selected demographic, clinical, biochemical and haematological parameters. ‘Pearson *r*’ was considered ‘high’ when it was > 0.70, ‘good’ when it was between 0.50 and 0.70, ‘fair’ if it was between 0.30 and 0.50 and ‘weak or no association’ if it was < 0.30 [28]. The accepted level of significance was *p* < 0.05.

## Results

### Participants

**Figure 1.**
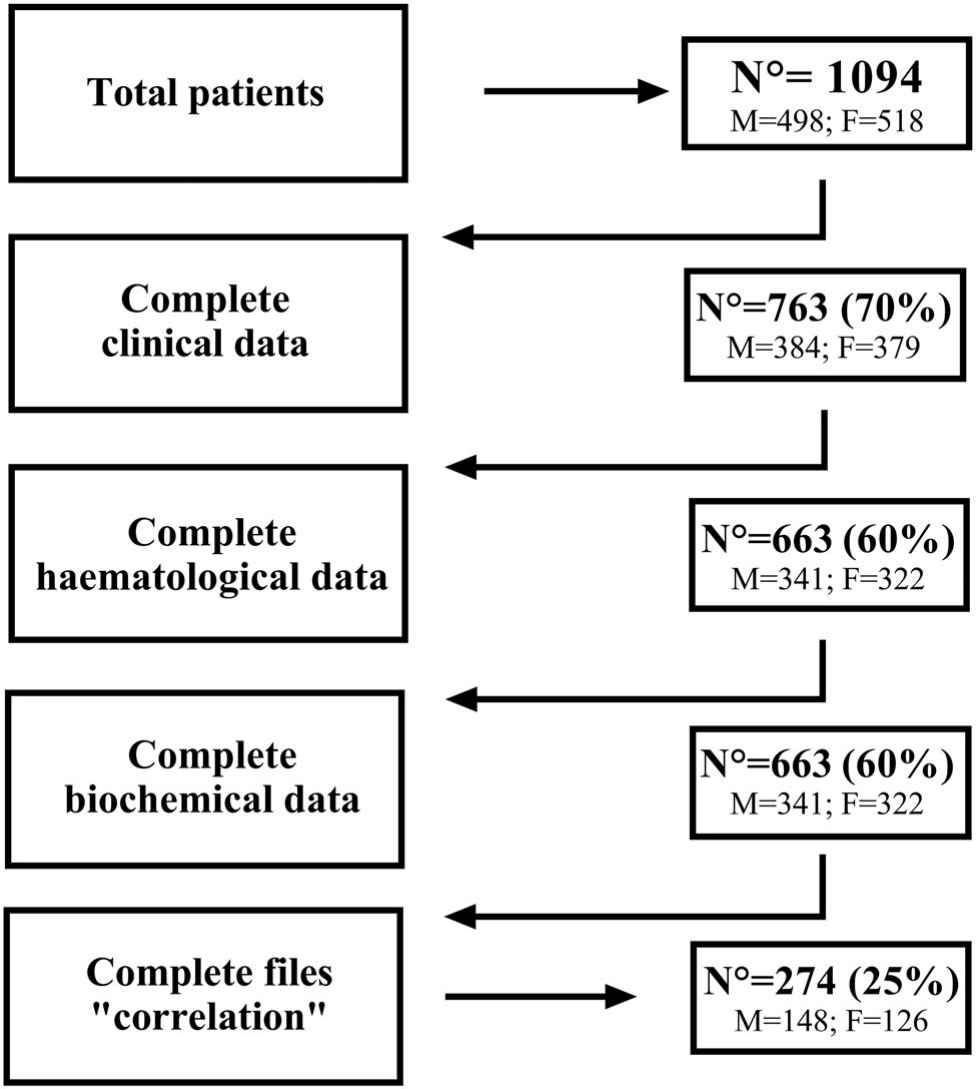
Number of participants, and missing data for each parameter. *N*: number of valid files; M: males; and F: females.

The overall sample included 1094 patients. However, since data were acquired under crisis conditions, some were missing, and not all the patients have complete files. Figure 1 shows the number of participants and missing data for each parameter.

### Univariate analysis

**Figure 2.**
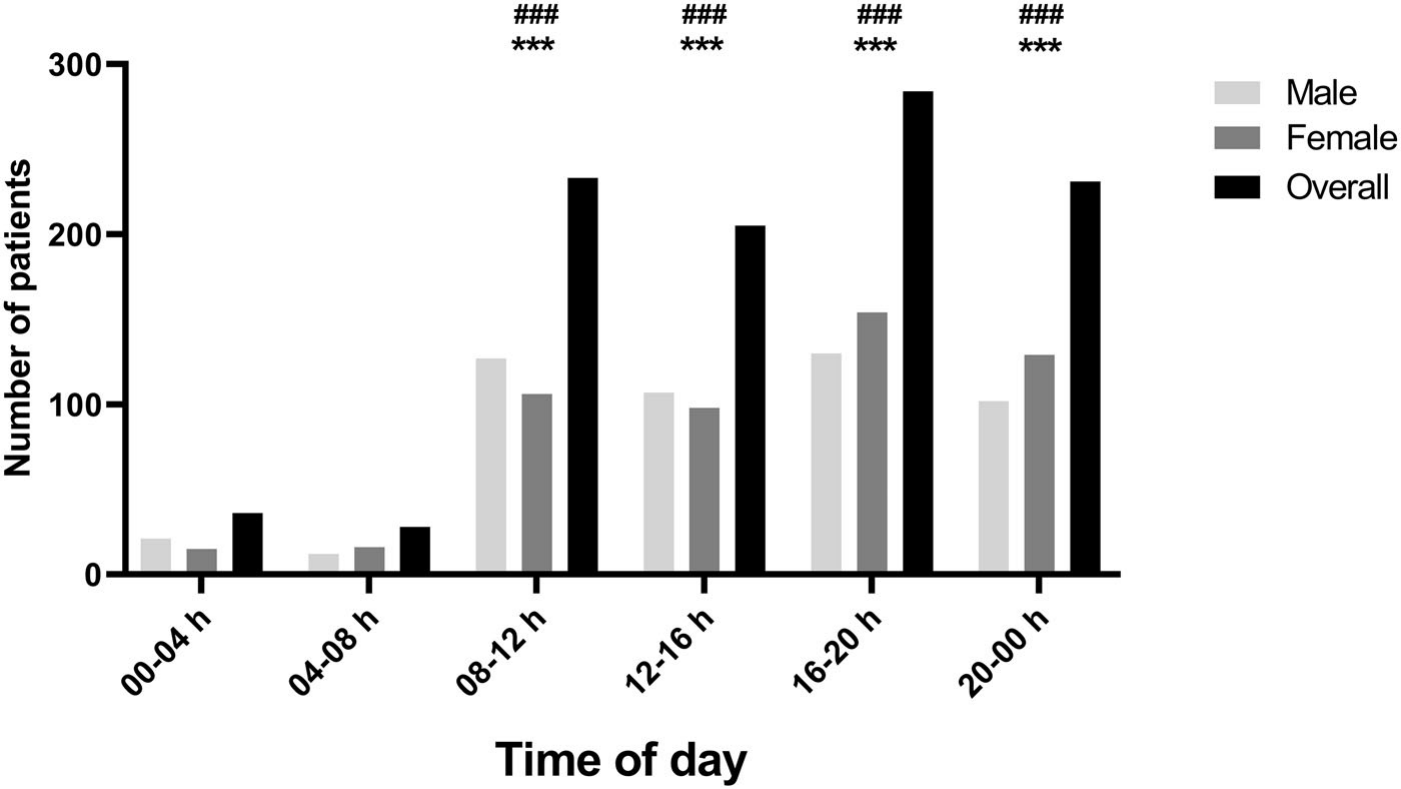
Patients’ distribution frequency per TOD (*n* = 1094). Data was processed using a One-way ANOVA with *** Means a significant difference compared to 00-04 h for male, female and overall. ^###^ Means a significant difference compared to 04-08 h for male, female and overall.

The power analysis for univariate analysis was a posteriori calculated and showed an observed power of 1. The number of COVID-19 patients attending the health care centre was higher between 08h00 and 00h00 compared to 00h00 and 08h00 (all *p* < 0.001) with no difference between sexes (Figure 2). CT, core body temperature, leukocytes and neutrophils were not affected by the TOD (Table 1). However, resting heart-rate and SpO_2_ were lower between 00h00-04h00 and heart-rate was higher at 20h00-00h00 compared to other TODs. Erythrocytes (at; 08h00-12h00) and platelets (at; 12h00-16h00) count were higher compared to other TODs. In addition, uraemia and glycaemia levels were higher between 00h00 and 04h00 compared to other TODs. Table 1 summed the univariate effects of TOD on the selected clinical, haematological, and biochemical data.

**Table 1.** Clinical, haematological and biochemical data at different TOD (in hours) in COVID-19 patients (*n* = 1094).

	Data (unit)	N°	00–04	04–08	08–12	12–16	16–20	20–00	*p* Value
Clinical data	Cycle-threshold (au)	1094	23.1 (6.1)	21.5 (6.3)	23.4 (5.8)	23.1 (5.8)	23.6 (6.2)	23.4 (5.7)	NS
	Temperature (°C)	763	36.9 (0.7)	37.2 (0.7)	37.2 (0.6)	37.3 (0.7)	37.3 (0.7)	37.2 (0.6)	NS
	Resting heart rate (bpm)	838	83.2 (13.4)*^abcde^	91.4 (16.9)	92.3 (15.4)	92.3 (15.1)	91.8 (14.5)	97.6 (18.7)*^eiklm^	<0.001
	SpO_2_ (%)	892	97.4 (1.6)*^abcde^	97.5 (1.8)	97.8 (1.5)	98.1 (1.3)	98.3 (1.4)	98.2 (1.3)	<0.001
Haematological data	Leukocytes (10^3^/μl)	663	5.6 (2.6)	5.9 (2.4)	6.1 (1.9)	5.9 (2.6)	5.8 (1.6)	6.1 (2.2)	NS
	Neutrophils (10^3^/μl)	663	3.3 (2.4)	3.6 (2.9)	3.3 (1.5)	3.9 (2.7)	3.1 (1.4)	3.2 (1.8)	NS
	Erythrocytes (10^6^/μl)	663	4.9 (0.7)	5.9 (2.5)*^afghi^	5.1 (0.6)	5.2 (0.6)	4.9 (0.7)	4.9 (0.7)	<0.001
	Thrombocytes (10^3^/μl)	663	203 (83)	210 (82)	260 (89)*^afj^	228 (72)	234 (64)	255 (66)	<0.001
Biochemical data	Urea (mmol/l)	663	6.3 (2.6)*^abcde^	4.7 (1.7)	4.3 (1.9)	3.8 (1.3)	3.2 (1.1)	4.2 (1.4)	<0.001
	Albumin (g/l)	663	40.9 (7.2)	41.3 (3.1)	41.4 (6.3)	42.1 (6.5)	43.1 (6.8)	52.1 (25.9)^*eiklm^	<0.001
	Glucose (mmol/l)	663	8.5 (4.4)*^abcde^	7.7 (3.1)*^ghi^	7.7 (3.9)	6.7 (2.9)	5.9 (3.1)	5 (0.7)	<0.001

Data were expressed as mean (SD).

*n*: total number of patients; *N*: number of patient with valid files (i.e. after excluding files with missing data); NS: not significant; SpO_2_: oxy-haemoglobin saturation.

*ANOVA^:^ significantly different from other TOD at *p* < 0.05. • 00–04 vs. ^a^04–08 or ^b^08–12 or ^c^12–16 or ^d^16–20 or ^e^20–00. • 04–08 vs. ^f^08–12 or ^g^12–16 or ^h^16–20 or ^i^20–00. • 08–12 vs. ^j^12–16 or ^k^20–00. • 12–16 vs. ^l^20–00. • 16–20 vs. ^m^20–00.

### Multivariate analysis

**Figure 3.**
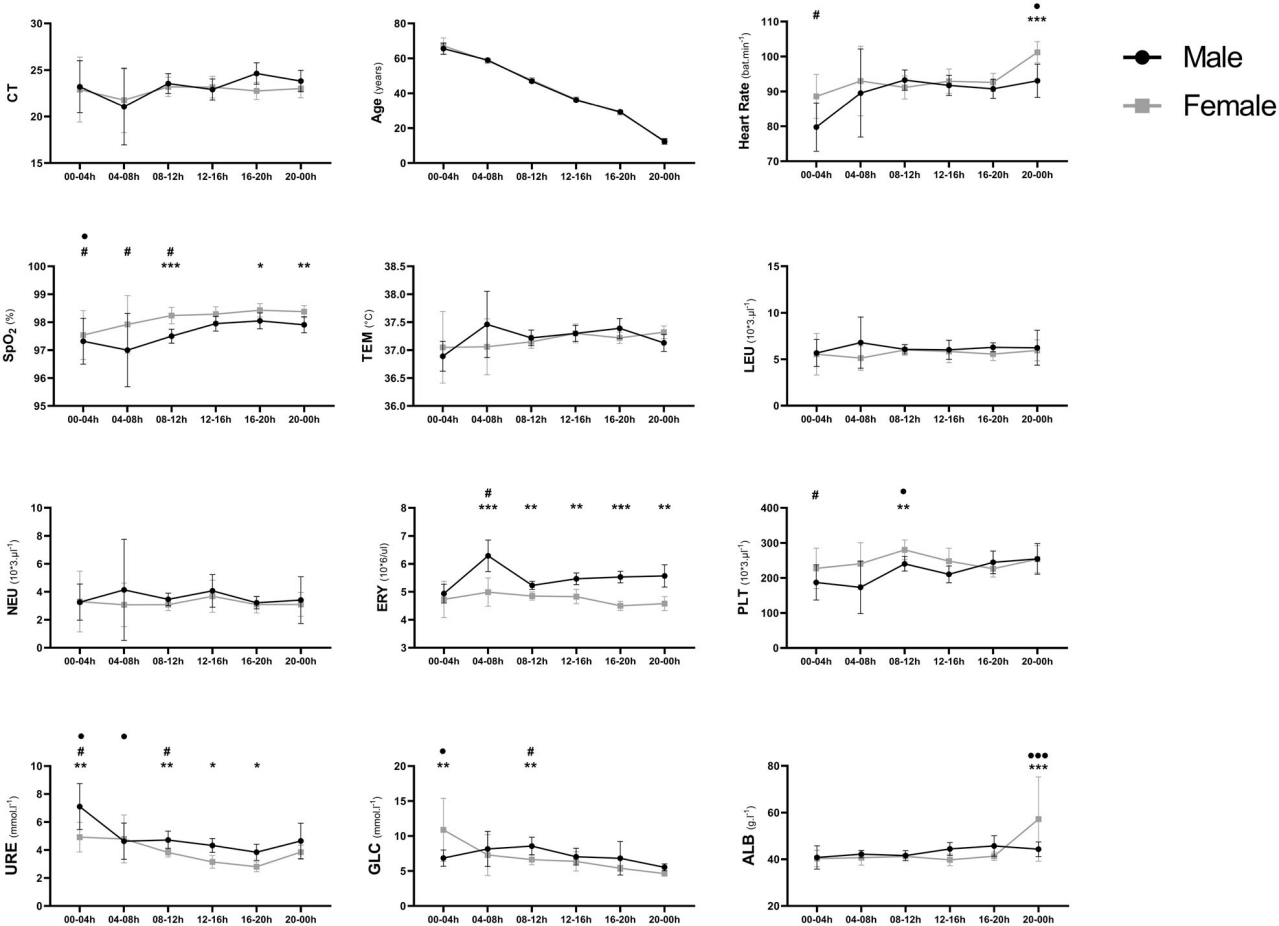
Male-Female comparison of different clinical, haematological and biochemical data at different TOD. Data was assessed using a two-way ANOVA followed by Bonferroni post-hoc test. *, ** and *** Significant sex-based difference at *p* < 0.05, *p* < 0.01 and *p* < 0.001. ^•^, ^••^ and ^•••^ Means significant TOD difference in females at *p* < 0.05, *p* < 0.01 and *p* < 0.001. ^#^, ^##^ and ^###^ means significant TOD difference in males at *p* < 0.05, *p* < 0.01 and *p* < 0.001.

The power analysis for multivariate analysis was a posteriori calculated and showed a strong observed power of 0.96. Male patients showed higher erythrocytes and uraemia compared to females (all; *p* < 0.05), and females showed higher SpO_2_ compared to males (all; *p* < 0.05) at different TODs. Mean and SD of different clinical, haematological, and biochemical data according to TOD and sex are presented in Figure 3.

### Correlation analysis

The power analysis for the correlation analysis was a posteriori calculated and showed an observed power of 0.92. The correlation was performed on 274 complete files. There was a near perfect reverse correlation between the age of patients and TOD of hospital admission (*r*=–0.97; *p* < 0.001). Pearson ‘*r*’ matrix is presented in Table 2.

**Table 2:** Correlation-coefficient (*r*) between different clinical, haematological and biochemical data (*n* = 274).

		Clinical data					Haematological data				Biochemical data		
				Age	CT	TEM	HR	SpO_2_	LEU	NEU	ERY	PLT	URE	ALB	GLC
Data	TOD	–0.97*^a^	0.04	0.07	0.16*^c^	0.14*^c^	0.01	–0.16	–0.06	0.06	–0.36*^b^	0.23*^c^	–0.27*^c^
Clinical data	Age		–0.03	–0.06	–0.19*^c^	–0.14*^c^	–0.02	0.11	0.06	–0.06	0.34*^b^	–0.27*^c^	0.26*^c^
	CT			–0.14*^c^	–0.16*^c^	0.02	0.22*^c^	0.21	0.01	0.25*^c^	–0.07	–0.05	–0.06
	TEM				0.31*^b^	–0.09*^c^	0.08	0.17	–0.02	–0.11	–0.04	0.07	0.06
	HR					–0.11*^c^	0.08	0.08	0.00	0.02	–0.14*^c^	–0.02	0.10
	SpO_2_						–0.14*^c^	–0.29*^c^	–0.14*^c^	0.07	–0.03	0.04	–0.13*^c^
Haematological data	LEU								0.01	0.39*^b^	–0.07	0.04	–0.02
	NEU									0.38*^b^	–0.02	–0.14	0.11
	ERY									–0.17*^c^	0.05	–0.03	0.10
	PLT										–0.14*^c^	–0.03	–0.05
Biochemical data	URE											–0.02	0.11
	ALB												–0.12

ALB: albumin; CT: cycle threshold; ERY: erythrocytes; HR: resting heart rate; LEU: leukocytes; NEU: neutrophils; PLT: platelets; SpO_2_: oxy-haemoglobin saturation; TEM: temperature; TOD: time of day; URE: urea.

*p < 0.05.

‘*r*’ was considered.

^a^High when it was > 0.70.

^b^Fair if it was between 0.30 and 0.50.

^c^Weak or having no association if it was <0.30.

## Discussion

The main outcome of this study was that hospital attendance in COVID-19 patients displays a diurnal variation, with patients attending hospital most in the active behavioural phase (between 08h00 and 00h00) compared to resting behavioural phase of the day (00h00-08h00). A strong negative correlation was found between the age of patients and the TOD of hospital attendance, with older patients attending the hospital earlier during the day (00h00-04h00). However, the TOD of hospital attendance was not affected by the sex of patients. In addition, COVID-19 infection upregulated the circadian rhythms of several haematological, clinical and biochemical parameters.

The current results showed that COVID-19 patients attend the hospital most during the active phase (08h00-00h00) compared to the resting phase (00h00-08h00). A recent study reported that SARS-CoV-2 positive tests were TOD dependent with a peak around 14h00 [15]. Although the former study reported on ∼86,000 patients from 130 different clinical locations and over 20 weeks, the reported TOD pattern showed similar trends as the current study. Therefore, the current findings validate the assumptions reported by McNaughton *et al.* [15]. In the same way, diurnal variation in the severity of local and systemic symptoms of common cold and influenza has been reported to be greater in the early morning [29]. Similarly, cough frequency in rhinorrhoea patients is more prominent during the initial hours after awakening from nocturnal sleep [12]. This could be consequent to symptoms aggravation during the previous night. It was reported that asthma is more severe during the night time [29], probably because of the infection-induced disruption in innate immunity [30]. It is well known that innate and adaptive immunities are under tight circadian control, with circulation peak occurring during the early night [30–34]. However, the current results showed that the circadian rhythms of neutrophils and leukocytes families were blunted in COVID-19 positive patients attending the hospital. In cases of viral infection, clock gene expression is disturbed due to systemic inflammation [35,36]. The proinflammatory cytokine interferon is released chronically during viral infection, which has the ability to interfere with circadian regulation [37]. This could indicate that SARS-Cov-2 infection affected clock gene expression, namely the brain and muscle ARNT-like 1, which dampened the diurnal variation of immune cells activities [36]. A near perfect reverse correlation (*r* = −97) was found between age and TOD in hospital attendance, with older patients attending the health care centre earlier during the day compared to younger patients. For instance, morningness preference (shift of rest/activity rhythm to the morning) is associated with advanced age [16,38], which could explain why older patients attend the health care centre at an earlier TOD compared to younger patients. Also, ageing is the most significant risk factor for developing severe outcomes and fatality in COVID-19 patients [2]. Indeed, developing severe COVID-19 outcomes could be related to the age-related immunosenescence [2], which could be defined as a chronic, low grade activation of proinflammatory process within the aged-organism [16]. The current data showed that oldest patients attended the care centre between 00h00 and 04h00. This could be explained by the increased asthma symptoms during the night [29] and the age-related decrease in physiological reserve in the respiratory system [2] that has aggravated COVID-19 symptoms, requiring immediate hospital attendance in older patients.

The current results showed that the CT (i.e. viral load) was unaffected by the TOD. Therefore, the assumption that COVID-19 hospital attendance is affected by the TOD could be somehow misleading. Contrarily, a recent study showed that the viral load was lower during the day in SARS-CoV-2 patients [15]. In fact, only female patients showed a trend towards lower CT values between 16h00 and 20h00, but this difference was not significant. These conflicting results might be explained by the sample size, which is larger from different locations and with a longer collection period in the above mentioned study [15]. Indeed, further research on larger sample size and different cohorts is required to confirm, or deny, the current results.

It has consistently been reported that core body temperature displays a strong circadian rhythmicity [39,40], which was blunted in the current study by COVID-19 infection. This could be explained by the infection-induced fever, which is associated with improved survival and resolution of infection [41]. In addition, COVID-19 infection shifted the circadian rhythms of resting heart-rate. Physiological resting heart-rate is higher during the morning [40,42] but in the current study, heart-rate was higher during the night (20h00-00h00) compared to the other TODs. Indeed, weak but highly significant correlations were found between CT on the one hand, and core body temperature and heart-rate on the other hand. In addition, SARS-Cov-2 infection shifted the circadian rhythms of uraemia, which was higher 00h00-04h00 compared to all other TODs. Contrarily, physiological uraemia was reported to be lower at this TOD (00h00-04h00) and higher between 08h00-14h00 [40]. The current finding showed that the circadian rhythm of glycaemia was upregulated by the COVID-19 infection only in female patients. Physiological glycaemia is higher during the beginning of the active phase of the day [43], which was true only for male patients in the current study (i.e. higher glycaemia between 08h00 and 12h00). However, glycaemia was higher between 00h00 and 04h00 in female patients. Although the origin of this upregulation is not clear, it could be associated with the inflammation-induced fever. Actually, it has been reported that a 1 °C increase in core body temperature requires a 10%–12% increase in metabolic rate [41]. Taken together, these findings strengthen the fact that the SARS-Cov-2 infection disrupted circadian rhythms regulation, probably *via* circadian gene expression channels [36,44]. Contrary to the strong relationship between the time of hospital attendance and the age of the patient, the TOD of hospital attendance was not related to the sex of the patients. Actually, the sex-based difference in SpO_2_ persisted in the current study, with females showing higher SpO_2_ compared to males, similarly to earlier reports [45]. Moreover, males showed higher erythrocytes level compared to females at different TODs, similarly to physiological haematocrit concentration reported elsewhere [46]. This could be indicative of a negligible effect of sex in regard to SARS-Cov-2 infection.

The current findings could be of relevance in the understanding of the temporal pattern of the COVID-19 pandemic. Patients attend health care centres at different TOD and for various reasons. Whether the presentation at a particular TOD has an effect on their overall outcome during the disease process is a key question. Identifying such times will help clinicians to prioritize, escalate, and manage such patients appropriately so that they receive the best possible outcome for their condition. Also, adjusting staffing numbers in health centres when more patients are present could be helpful in dealing more effectively as well as help improve efficiency whilst dealing with the challenges of this pandemic.

To the authors’ finest knowledge, this is the first study to investigate the diurnal variations in symptoms of COVID-19 patients. Diurnal variation in symptoms of cold and influenza has been reported in the literature [11]. Although the current results showed that COVID-19 patients’ hospital admission is influenced by the TOD, the findings do not conclusively support that COVID-19 symptoms follow the same circadian pattern. First, the CT was not influenced by the TOD. Second, the time of viral infection may have affected the time of clinical presentation in these patients [11], which is beyond the scope of this study. Besides, the relatively long incubation period of SARS-CoV-2 makes it difficult to identify retrospectively the onset time of infection. Indeed, the causal relationship between TOD and COVID-19 remains unclear and still to be explored. Furthermore, a longitudinal follow up of patients’ vitals and the development of their symptoms would be very helpful to a better understanding of this pandemic. No exclusion criteria were applied in the current study, which could limit the current assumptions. In addition, there was no control group (e.g. patient attending the healthcare centre for other reasons) in the current study. Indeed, the lack of comparison could lead to biased conclusions. Thus, further studies on larger scales could generate a better understanding of the relationship.

## Conclusion

COVID-19 patients’ attendance at the health care centre was found to be associated with the rest/activity pattern. However, the CT was unaffected by the TOD, which limits the conclusion that COVID-19 symptoms’ gravity is modulated by the TOD. Older patients attend the health care centre earlier during the day compared to younger patients. Furthermore, COVID-19 dampened the circadian variation in core body temperature, circulating leukocytes and neutrophils and shifted the circadian variation of heart-rate and uraemia.

## Data Availability

All data analysed and reported in this study are available from the first author on reasonable request.
